# Transforming heart transplantation care with multi-omics insights

**DOI:** 10.1186/s12967-025-06772-0

**Published:** 2025-07-01

**Authors:** Zhengbang Zou, Jianing Han, Zhiyuan Zhu, Shanshan Zheng, Xinhe Xu, Sheng Liu

**Affiliations:** National Clinical Research Center of Cardiovascular Diseases, National Center for Cardiovascular Diseases, Fuwai Hospital, Chinese Academy of Medical Sciences and Peking Union Medical College, Beijing, 100037 China

**Keywords:** Heart transplantation, Multi-omics, Biomarkers, Cell-free DNA, Gene expression profiling, MicroRNAs, Machine learning

## Abstract

Heart transplantation (HTx) remains the definitive treatment for patients with end-stage heart disease. Despite the number of HTx performed annually in worldwide continues to increase, complications of HTx still impact the quality of life and long-term prognosis, including rejection, infection, and allograft dysfunction. Endomyocardial biopsy remains the gold standard for monitoring cardiac allograft rejection post-heart transplantation, yet its invasiveness and interobserver error in histologic grading necessitate the development of novel noninvasive biomarkers to elucidate rejection mechanisms and progression. Cardiac allograft vasculopathy, a critical determinant of long-term outcomes, is challenging to detect early via intravascular ultrasound, underscoring the potential of plasma biomarkers for disease surveillance. Omic technologies usually refers to the application of multiple high-throughput screening technologies enabling comprehensive analysis of biological systems at a molecular level. Multi-omics technologies, including genomics(donor-derived cell-free DNA), transcriptomics(microRNAs panels, gene expression profiling), proteomics(cell signaling molecule), and metabolomics(ex situ heart perfusion), have demonstrated significant promise in post-transplant monitoring. These approaches provide personalized risk stratification and mechanical insights into cardiac allograft rejection, primary graft dysfunction, and cardiac allograft vasculopathy. Single–cell omics technologies and machine learning algorithms further resolve cellular heterogeneity and improve predictive modeling, thereby enhancing the clinical translatability of multi-omics data. This comprehensive review synthesizes these advances and highlights the transformative potential of integrating multi-omics with advanced analytics to achieve precision monitoring and therapy in HTx, ultimately improving long-term patient outcomes.

## Introduction

Heart transplantation (HTx) continues to be the definitive treatment for patients with end-stage heart disease of varying etiologies, with more than 6,000 heart transplants performed annually worldwide [1]. Nevertheless, the long-term survival and quality of prognosis are constrained by post-HTx complications, including cardiac allograft rejection (CAR), cardiac allograft vasculopathy (CAV) and primary graft dysfunction (PGD) [1, 2]. Current surveillance of CAR relies heavily on invasive endomyocardial biopsies (EMBx), which are associated with a complication rate of 2.3%, age restrictions, and potential sampling errors [3–5]. Invasive coronary angiography (ICA) and intravascular ultrasound (IVUS) are diagnostic procedures for CAV, with significant costs and potential complications, such as bleeding, infection, and acute kidney injury, which are not suitable for all recipients [6, 7]. PGD, an early idiopathic ventricular dysfunction after HTx. The RADIAL score is the only effective scoring system that can predict the clinical risk of PGD, an early idiopathic ventricular dysfunction after HTx; however, it lacks accuracy [8].

Omics technologies are becoming increasingly accessible and can be used to identify novel biomarkers while revealing the underlying biology of various diseases. These technologies have emerged as transformative tools for identifying potential circulating biomarkers for rejection monitoring and post-transplant injuries [9–11]. Leveraging multi-omics methodologies, which encompass genomics, transcriptomics, proteomics, and metabolomics analyses, has significantly advanced our understanding of the pathophysiological mechanisms governing allograft complications in HTx [12–14]. Single-cell and multi-omics analyses have gained considerable attention in the pursuit of clinical transformations because of their ability to dissect cellular heterogeneity and capture dynamic molecular changes that may serve as potential therapeutic targets [15, 16]. Machine learning, with its capacity to process extensive datasets and identify intricate patterns, has demonstrated promise for the development of predictive models for adverse events following transplantation [17]. Despite standard combinations of immunosuppressants administered to HTx recipients, research and innovations in immunosuppressive therapy over the past two decades remain lacking. This stagnation is partly attributed to a poor understanding of biomarkers associated with immunosuppression. Identifying actionable risk biomarkers for post-transplant adverse events could facilitate the personalization of conventional one-size-fits-all post-transplant management paradigms. These approaches enhance the survival rates and reduce the incidence of adverse events in HTx recipients. With the application of these technologies in clinical settings, a deeper understanding of individualized factors that affect prognosis, such as recipient age, sex, and race, should be considered [9, 18, 19]. 

This state-of-the-art review aims to systematically summarize the applications of multi-omics technologies in HTx, and to describe the value and future directions of these technologies in the clinical transformation of heart transplant care.

### Omics applications in CAR and early graft injury

#### Prediction of CAR and early graft injury

Genomics analysis has identified thousands of DNA sequences that are widely used in HTx (Table 1). Donor-derived cell-free DNA (dd-cfDNA) serves as a non-invasive biomarker for CAR and includes both acute cellular rejection (ACR) and antibody-mediated rejection (AMR). This approach is recommended by the International Society for Heart and Lung Transplantation (ISHLT) and the American Society of Transplantation (AST) [20–22]. The Genomic Research Alliance for Transplantation (GRAfT) conducted a multi-center prospective study involving 165 transplant recipients, where genetic profiling was used to determine donor-recipient single nucleotide polymorphisms (SNPs). Unbiased shotgun sequencing was employed to quantify the proportion of plasma dd-cfDNA relative to total cfDNA (%dd-cfDNA). The study confirmed that an elevated percentage of dd-cfDNA was correlated with both the severity of CAR and early graft injury (p < 0.001).When the %dd-cfDNA threshold was set at ≥ 0.25, the area under the receiver operating characteristic (AUROC) curve ranged from 89 to 95%, demonstrating excellent sensitivity and specificity, with a negative predictive value (NPV) reaching 99%. Furthermore, 81% of myocardial EMBx can be avoided when % dd-cfDNA level remains < 0.25 [10].

**Table 1:** Application of genomics in HTx

Author	Study type	Technology	Recipients characteristics	Etiology	Sample characteristics	Conclusion	Ref
Palak Shah et al. (2024)	Multi-center prospective cohort study	Unbiased shotgun sequencing; ddPCR	Recipient, n = 148 (Black, n = 65; White, n = 83); Median age, y (IQR): 56.0 (45.0–62.0); Male patient (%): 70.0; Female patient (%): 30.0; Mean follow-up time: 4.4 ± 1.3y	Ischemic cardiomyopathy (%): 28.0; Nonischemic cardiomyopathy (%): 60.0; Other (%): 12.0	Sample type: EMBx; plasma; Sample number: plasma, n = 1551 (dd-cfDNA); plasma, n = 1160 (cfmtDNA)	Increased dd-cfDNA and cfmtDNA were related to the high incidence of CAR and worse clinical outcome in Black patients after HTx, cfmtDNA may be an optimal mediator for enhancing alloimmune response and clinical outcome Improving Black patients prognosis after HTx, and promoting personalized immunotherapy for different races	[25]
Rami Alharethi et al. (2024)	Multi-center prospective study	Targeted NGS (AlloSure); qRTPCR	Recipient, n = 69; Mean age, y (SD): 58.6 (13.7); Male patient (%): 66.7; Female patient (%): 33.3	–	Sample type: plasma; Sample number: Plasma, n = 101; Sample collecting time: 8.7 ± 7.1y after HTx	Dd-cfDNA was not associated to the presence or severity of CAV, and may not be a reliable non-invasive biomarker for CAV monitoring	[6]
Ersilia M. DeFilippis et al. (2024)	Multi-center prospective study	Shotgun sequencing	Recipient, n = 151; Median age, y (IQR): 55.0 (48.0–62.0); Male patients (%): 68.0; Female patients (%): 32.0; Median follow-up time: 329d	CAD (%): 12.2; Dilated myopathy, idiopathic (%): 32.4; Other (%): 55.4	Sample type: EMBx; plasma; Sample number: plasma, n = 1119; Sample collecting time: ≥ 28 d after HTx	No-significant gender difference in dd-cfDNA level during the rejection period. Different genders may adopt similar diagnostic thresholds for rejection monitoring	[19]
Amit H. Alam et al. (2024)	Single-center prospective observational study	AlloSure	Recipient, n = 37 (CMV +, n = 12; CMV-, n = 25); Median age, y (IQR): 60.5 (54.8–66.7); Male patients (%): 73; Female patients (%): 27	Ischemic cardiomyopathy (%): 35.0	Sample type: EMBx; plasma;	Dd-cfDNA monitoring should consider the impact of active CMV infection, and it is essential to further verify the connection between CMV and dd-cfDNA	[37]
Jens Böhmer et al. (2023)	Single-center prospective cohort study	dPCR	Recipient, n = 52 (pediatric, n = 11; adult, n = 41); Median age, y (range): 52.5 (1.0–68.0); Male patients (%): 69.0; Female patients (%): 31.0; Median follow-up time: 1y	-	Sample type: EMBx; plasma; Sample number: plasma, n = 557; Sample collecting time: < 14 d after HTx	Reporting only %dd-cfDNA may not be reliable and did not consider rd-cfDNA. Quantifying absolute levels adds important value to the differentiation between ongoing graft damage and quiescent situations As compared with females, males had significantly lower levels of rd-cfDNA	[34]
Kaushik Amancherla et al. (2023)	Multi-center retrospective study	Targeted NGS	Recipient, n = 479; Cohort 1 (n = 301) CHIP, median age, y (range): 59.0 (52.0–65.0); Male patients (%): 79.0; Female patients(%): 21.0; No-CHIP, median age, y (range): 49.0 (36.0–59.0); Male patients (%): 73.0; Female patients (%): 27.0; Cohort 2 (n = 178) CHIP, median age y (range): 60.0 (52.0–66.0); Male patients (%): 69.0; Female patients (%): 31; No-CHIP, median age, y (range): 49.0 (36.0–59.0); Male patients (%): 69.0; Female patients (%): 27.0; Median follow-up time: cohort 1 18y; cohort 2, 5.1y	Cohort 1 CHIP ischemic cardiomyopathy (%): 62.0; No-CHIP ischemic cardiomyopathy (%): 40.0; Cohort 2 CHIP ischemic cardiomyopathy (%): 29.0; No-CHIP ischemic cardiomyopathy(%): 23.0	Sample type: plasma; Sample number: plasma, n = 479; Sample collecting time: a median time of 2.3y before orthotopic HTx	The presence of CHIP mutations was not associated with an increased risk of CAV or posttransplant mortality CHIP may not be a significant driver of CAV	[48]
Nicholas Rodgers et al. (2023)	Single-center retrospective, observational study	Shotgun sequencing	Recipient, n = 112; Median age, y (IQR): 60.0 (46.8–65.3); Male patients (%): 77.7; Female patients (%): 22.3	Nonischemic cardiomyopathy (%): 67.9; Ischemic cardiomyopathy (%): 26.8; Congenital (%): 2.7; Retransplant (%): 2.7	Sample type: EMBx; plasma; Sample number: plasma, n = 428 (paired GEP, n = 316); Sample collecting time: median time from HTx to EMBx was 114 d (IQR, 65-200d); ≥ 28 d after HTx, < 14 d before EMBx SNP detecting; > 55 d after HTx GEP detecting	No-significant difference between standard and expanded SNP assays in detecting CAR, the application of both in clinical practice should be carefully considered, including threshold selection Using dd-cfDNA in place of GEP testing improved specificity without change in sensitivity Prospective controlled studies to address how to best implement dd-cfDNA testing into clinical practice are needed	[20]
Timea Teszak et al. (2023)	–	Targeted NGS (AlloSeq)	Recipient, n = 26	–	Sample type: plasma; Sample number: plasma, n = 90	Analyzing dd-cfDNA at the local level was feasible, considerably reducing the frequency of invasive surveillance EMBx	[11]
Marta Jiménez-Blanco Bravo et al. (2022)	Single-center prospective, observational study	NGS	Recipient, n = 94; Median age, y (IQR): 57.0 (50.0–67.0); Male patients (%): 67.0; Female patients (%): 33.0	–	Sample type: plasma	Dd-cfDNA did not perform as a useful biomarker to avoid surveillance coronary angiograms for CAV diagnosis	[49]
Paul J. Kim et al. (2022)	Multi-center observational study	Available SNP-based massively multiplexed-PCR; NGS	Recipient, n = 223; Median age, y (IQR): 54.0 (41.0–63.0); Male patients (%): 73.0; Female patients (%): 27.0	Nonischemic cardiomyopathy (%): 65.4; Ischemic cardiomyopathy (%): 23.3; Congenital (%): 4.5; Retransplant (%): 1.8; Other/Missing (%): 4.9	Sample type: EMBx; plasma; Sample number: plasma, n = 811 (paired EMBx); Sample collecting time: ≥ 28 d after HTx	A clinically available SNP-based massively multiplexed-PCR dd-cfDNA assay detects CAR in HTx recipients with good accuracy and holds promise as a noninvasive test for CAR The implications of elevated dd-cfDNA in patients with left ventricular dysfunction in the absence of CAR requires further study The content of dd-cfDNA (cp/mL) as a marker may be superior to %dd-cfDNA, a “two-threshold” algorithm was employed, which combines a cutoff for dd-cfDNA fraction with a cutoff for absolute quantity of dd-cfDNA	[27]
Fernando L. Scolari et al. (2022)	Single-center retrospective study	Targeted Error-corrected sequencing	Recipient, n = 127; Median age, y (range): 53.0 (30.0–76.0); Male patients (%): 77.0; Female patients (%): 23.0; Median follow-up time: 3.2 ± 2.6y	Ischemic cardiomyopathy (%): 22.0; Nonischemic cardiomyopathy (%): 71.0; Congenital (%): 7.0	Sample type: EMBx; plasma; Sample number: plasma, n = 127; Sample collecting time: median 200 d (IQR = 428) before HTx; median 114 d (IQR = 624) after HTx	CH was not associated with ACR, CMV infection or malignancies Prospective studies are warranted to assess CH at sequential timepoints, as this may also be a potential therapeutic target to augment post-transplant clinical course	[50]
Jeroen G.H.P. Verhoeven et al. (2022)	Single-center observational study	ddPCR	Recipient, n = 15; Mean age, y (range): 49.0 (18.0–63.0); Male patients (%): 60.0; Female patients (%): 40.0	–	Sample type: EMBx; plasma; Sample number: plasma, n = 113 (paired EMBx); Sample collecting time: plasma, before EMBx (< 15 min) and after EMBx (< 15 min); paired EMBx 7-509d after HTx	The EMBx procedure caused iatrogenic injury to the allograft that results in an increase in %dd-cfDNA and dd-cfDNA concentrations For the assessment of dd-cfDNA as marker for CAR, collection of plasma samples before the EMBx procedure is essential	[32]
Aasim Afzal et al. (2022)	Single-center retrospective, observational study	AlloSure	Recipient, n = 35; Median age, y (range): 62.1 (55.8–65.7) Male patients (%): 86.0 Female patients (%): 14.0 Follow-up time: 1y	Ischemic cardiomyopathy (%): 46.0	Sample type: plasma	Clinical characteristics to distinguish groups of patients with elevated dd-cfDNA result to include ACR (1R) with CMV viremia, non-CMV infection As many as 23% of patients with elevated AlloSure values had right ventricular dysfunction, graft injury markers may be higher in patients with persistent right ventricular dysfunction	[29]
Megan Kamath et al. (2022)	Single-center retrospective, pilot study	AlloSure; AlloMap	Recipient, n = 72; Mean age, y (SD): 49.1 (14.3); Male patients (%): 62.5; Female patients (%): 37.5; Median follow-up time: 480d	Nonischemic cardiomyopathy (%): 69.4; CAD (%): 20.8; Retransplant (%): 9.7	Sample type: plasma	Increased variability of dd-cfDNA in HTx was associated with both mortality risk and the presence of DSA The longitudinal data in the interpretation of AlloMap/AlloSure scores in this population was valuable	[51]
Brian Feingold et al. (2021)	Single-center prospective study	qRTPCR	Recipient, n = 58; Median age, y (range): 14.8 (8.4–18.3); Male patients (%): 66.0; Female patients (%): 34.0; Median follow-up time: 8.7 m (4.2–15.0)	–	Sample type: EMBx; plasma; Sample collecting time: median 6.0y (2.2–11.2) after HTx	It is feasible to replace the selected EMBx with dd-cfDNA assessment in pediatric HTx	[12]
Sean Agbor- Enoh et al. (2021)	Multi-center prospective study	Unbiased paired-end shotgun sequencing	Recipient, n = 165; Mean age, y (range): 53.0 (20.0–70.0); Male patients (%): 72.0; Female patients (%): 28.0; Median follow-up time: 17.7 m	Nonischemic cardiomyopathy (%): 53.0; Ischemic cardiomyopathy (%): 22.0; Hypertrophic cardiomyopathy (%): 1.0; Restrictive cardiomyopathy (%): 1.0; Myocarditis (%): 1.0; Other (%): 22.0	Sample type: EMBx; plasma; Sample number: EMBx, n = 1392; plasma, n = 1834; Sample collecting time: 7, 14, 28, 45 d after HTx	Monitoring with dd-cfDNA demonstrated excellent performance characteristics for both ACR and AMR and led to earlier detection than the EMBx-based monitoring After day 28 of surgery, heart transplant recipients could be monitored for CAR using %dd-cfDNA levels; specifically, if the levels remain < 0.25%, surveillance biopsies would no longer be required Differences in the pathogenesis of AMR and ACR may lead to differences in %dd-cfDNA levels	[10]
Marc E. Richmond et al. (2020)	Multi-center prospective, observational study	qRTPCR	Recipient, n = 241 (pediatric, n = 146; adult, n = 95); Mean age, y (SD): 23.7 (23.6); Male patients (%): 61.4; Female patients (%): 38.6	–	Sample type: EMBx; plasma; Sample number: whole blood, n = 564; plasma, n = 60 (paired EMBx, n = 624)	AUROC could be used to determine the optimal DF threshold, and 0.3% threshold and 80% NPV could distinguish ACR ≥ 1R and pAMR ≥ 1 Sample preparation is crucial to the accuracy of %dd-cfDNA detection, and the plasma protocol is superior to the whole blood protocol	[18]
Kiran K. Khush et al. (2019)	Multi-center prospective, observational study	AlloSure	Recipient, n = 443; Mean age, y (SD): 54.0 (12.0) Male patients (%): 74.0; Female patients (%): 26.0	Ischemic cardiomyopathy (%): 31.0 Nonischemic cardiomyopathy (%): 50.0 Congenital (%): 3.0; Multiple (%): 2.0; Other (%): 12.0; Retransplant (%): 1.0	Sample type: EMBx; plasma; Sample number: plasma, n = 841 (paired EMBx, n = 841); Sample collecting time: 55d-5y after HTx	%dd-cfDNA is useful for CAR screening, including ACR and AMR, suggests early graft dysfunction According to the results of dd-cfDNA detection, more effective immunosuppression could be administered to recipients at higher risk of CAR, whereas the side effects and toxicities of these medications could be avoided in stable patients	[26]
J. Beck et al. (2015)	Single-center prospective study	ddPCR	KTx recipient, n = 300; HTx recipient, n = 80	–	Sample type: EMBx; plasma	Dd-cfDNA noninvasiveness enables the monitoring recipients at intervals that are desired to catch rejections at early actionable stages to prevent full-blown rejection, and minimize immunosuppression	[33]
Iwijn De Vlaminck et al. (2014)	Single-center prospective cohort study	Shotgun sequencing GTD	Recipient, n = 65 (pediatric, n = 21; adult, n = 44); Adult mean age, y (range): 50.0 (20.0–69.0); Pediatric mean age, y (range): 8.0 (0–19.0); Male patients (%): 60.0; Female patients (%): 40.0	Ischemic cardiomyopathy (%): 10.8; Dilated cardiomyopathy (%): 46.2; Congenital (%): 13.8; Graft vasculopathy/retransplantation (%): 1.5; Hypertrophic cardiomyopathy (%): 12.3; Other (%): 13.8	Sample type: EMBx; plasma; Sample number: EMBx, n = 356; plasma, n = 565; Sample collecting time: 1 d, 7 d, > 14 d after HTx	the GTD approach was informative for detecting CAR and that GTD had the potential to complement or replace existing biopsybased surveillance approaches The GTD was not able to distinguish graft damage from AMR versus ACR, may require follow-up testing, such as biopsy or measurement of donor-specific anti-HLA antibodies, if rejection was determined	[4]

*PCR* polymerase chain reaction, *ddPCR* droplet digital PCR, *IQR* interquartile range, *y* year, *EMBx* endomyocardial biopsy, *dd-cfDNA* donor-derived cell-free DNA, *cfmtDNA* cell-free mitochondrial DNA, *HTx* heart transplantations, *NGS* next generation sequencing, *AR* acute rejection, *qRTPCR* quantitative real-time PCR, *SD* standard deviation, *CAV* cardiac allograft vasculopathy, *d* day, *CAD* coronary artery disease, *CMV* cytomegalovirus, *dPCR* digital PCR, *rd-cfDNA* recipientderived cell-free DNA, *CHIP* clonal hematopoiesis of indeterminate potential, *GEP* gene-expression profiling, *SNP* single nucleotide polymorphism, *CH* clonal hematopoiesis, *DSA* donor specific antibody, *m* month, *AMR* antibody-mediated rejection, *ACR* acute cellular rejection, *AUROC* area under the receiver operator characteristic curve, *NPV* negative predictive value, *DF* donor fraction, *KTx* kidney transplantation, *GTD* genome transplant dynamics, *HLA* human leukocyte antigen

Informative SNPs refer to instances in which the recipient has a homozygous genotype (two alleles with the same nucleotide) that differs from the donor’s genotype, which may be either a heterozygous or homozygous variant. These SNPs can be used to distinguish between donor and recipient DNA [4, 23, 24]. Whole-genome sequencing, based on informative SNPs, allows for the precise quantification of dd-cfDNA and provides strong evidence that an elevated %dd-cfDNA is associated with CAR [25]. However, pre-transplant DNA genotyping to identify SNPs increases the complexity of non-invasive screening. To address this, multiplex polymerase chain reaction (PCR) targeting SNPs can be used without prior genotyping of the donor or recipient, enabling the quantification of dd-cfDNA through next-generation sequencing (NGS) based on informative SNPs [18, 26]. However, pre-transplant DNA genotyping to identify SNPs increases the complexity of non-invasive screening. A clinically available massively multiplex PCR platform was used to amplify plasma cfDNA from 223 HTx patients, targeting over 13,000 SNPs to maximize the number of informative SNPs. This approach, combined with NGS, detected CAR with sensitivity of 78.5%, specificity of 76.9%, and a NPV of 97.3% when the %dd-cfDNA was ≥ 0.15, demonstrating good accuracy [27]. Interestingly, merely increasing the number of SNPs may not improve cfDNA quantification or dd-cfDNA detection performance. The impact of a higher number of informative SNPs on diagnostic benefit remains to be validated in multi-center prospective studies [20].

The complexity and cost of shotgun whole-genome sequencing limits its widespread adoption as a clinically relevant monitoring tool. Targeted quantification of dd-cfDNA, although a faster and more efficient monitoring method, requires pretransplant genotyping. AlloSure, a NGS method targeting highly polymorphic SNPs, enables dd-cfDNA quantification without the need for donor or recipient genotyping [26, 28, 29]. The Donor‐Derived Cell‐Free DNA-Outcome AlloMap Registry (D‐OAR) demonstrated that AlloSure aids in the early detection of CAR, with good specificity and NPV at a 0.2% threshold [26]. AlloSeq, a commercialized targeted NGS dd-cfDNA detection method, has shown reliability and reproducibility in distinguishing patients who experience rejection at different post-HTx time points from those who do not and can avoid 88% of EMBx for monitoring [11].

In clinical monitoring of heart HTx, NGS is limited by high detection costs and long turnaround times. Additionally, the abundance of dd-cfDNA in HTx recipients is low, and requires more sensitive quantification techniques [30, 31]. Digital droplet PCR (ddPCR), as a rapid and cost-effective method, detects minute genetic variations in donor and recipient DNA to quantify dd-cfDNA, and has been shown to identify early rejection in HTx recipients [32, 33]. Owing to the high variability of recipient cfDNA, dynamic monitoring of rejection is challenging, and quantifying the absolute levels of dd-cfDNA using ddPCR may serve as a better indicator than %dd-cfDNA [32]. A prospective cohort study using ddPCR and SNPs genotyping with target-specific pre-amplification-quantified cfDNA concentration. The study found that %dd-cfDNA was significantly elevated in CAR patients (p < 0.0001), with an AUROC of 0.78 and a sensitivity of 92% at a threshold of 0.1. Similarly, absolute levels of dd-cfDNA were significantly elevated during rejection (p = 0.0001), with an AUROC of 0.75 and sensitivity of 92% at a threshold of 7.5 copies/mL. This demonstrated a detection performance similar to that of %dd-cfDNA [34]. Relying solely on %dd-cfDNA may be unreliable, as it does not account for variations in dd-cfDNA, and %dd-cfDNA only identifies the rejection status at specific post-transplant time points. The"dual biomarkers with dual thresholds"approach might be feasible, combining the %dd-cfDNA threshold with an absolute level threshold for dd-cfDNA to distinguish ongoing graft injury from a stable state [27, 35, 36]. It is important to note that both ddPCR and NGS genomics molecular testing methods yield similar results, but with significant differences in turnaround time and cost [24, 30, 33]. There is no consensus on which quantification technique is more suitable for dd-cfDNA detection, and the lack of comparative data on their advantages and disadvantages indicates that clinical decisions are likely to be driven by the specific monitoring requirements and objectives of transplantation.

However, dd-cfDNA cannot serve as a specific biomarker for CAR, as its levels may also increase in cases of non-rejection-related injuries. For example, in HTx patients without rejection, the %dd-cfDNA level was significantly higher during active cytomegalovirus (CMV) infection (p < 0.001). Large-scale trials are needed to verify the effect of CMV or other complications on dd-cfDNA levels [37]. Because dd-cfDNA levels increase before the appearance of histological features of rejection, the results are often considered false positives, leading to a reduced positive predictive value (PPV) of dd-cfDNA testing [10], which hinders its use as a standard diagnostic tool for rejection. Conducting new biomarker studies, such as those focusing on cell-free mitochondrial DNA, may address the limitations of relying on a single diagnostic standard [38].

Metabolomics has the potential to identify the occurrence and development of early functional impairment after transplantation. Tarazón et al. analyzed serum and EMBx samples from 49 non-rejection HTx patients and 78 CAR patients and found that the levels of sarcoplasmic reticulum Ca^2^⁺-ATPase were decreased in CAR patients; this reduction was correlated with the severity of rejection, with an AUROC of 0.804 (p < 0.0001) [39]. The ability of these metabolites to serve as CAR biomarkers was validated in a rat ectopic heart transplant model. Lin et al. used liquid chromatography-mass spectrometry (LC–MS) to analyze blood and EMBx samples from rats at different time points. Four common differential metabolites, D-tagatose, choline, C16 sphingosine, and D-glutamine, each demonstrated an AUROC greater than 0.90 in identifying CAR [40]. These metabolites were combined into a diagnostic panel to achieve an AUROC of 0.983 for distinguishing CAR. In another study, Gas Chromatography/Time-of-Flight Mass Spectrometry (GC/TOFMS) was used to analyze the plasma metabolite levels in rats with ectopic heart transplants and rats treated with rapamycin. Proline, glycine, serine, phenylalanine, and isocitrate could distinguish CAR with AUROC exceeding 0.85. Further studies are required to validate the predictive ability of these amino acids as biomarkers for CAR in clinical cohorts [41]. Importantly, lower levels of leucine/isoleucine and long-chain acylcarnitine have been shown to be significantly associated with moderate-to-severe PGD (odds ratio [OR]: 0.42, OR: 0.50). These metabolites may serve as important early predictive markers of functional impairment after HTx [42].

Analysis of ex situ heart perfusion (ESHP) fluid before HTx aids in exploring the early functional status of the graft and the heart utilization pattern of energy metabolic substrates. Clinically, lactate levels have become an important standard for evaluating donor organ quality during ESHP, and lactate metabolism may be a significant pathway for energy acquisition by the donor heart. However, in the first metabolic analysis of human HTx pre-ESHP fluid samples, neither changes in lactate nor troponin I (TnI) levels (OR: 1.11, 95% confidence interval CI 0.34–3.64; OR: 0.91, 95% CI 0.48–1.72), nor end-of-run lactate or TnI levels (OR: 1.49, 95% CI 0.69–3.22; OR: 1.23, 95% CI 0.84–1.80) were associated with PGD [42]. In addition, Hautbergue et al. found that lactate levels in the myocardial tissue remained stable throughout the ESHP process, suggesting that lactate metabolism may not be the primary metabolic change during the early stages of HTx [43]. In contrast, LC–MS combined with GC/TOFMS analysis of pre-transplant ESHP fluid showed that branched-chain amino acids leucine/isoleucine, ketones, 3-hydroxybutyrate, and non-esterified fatty acids were rapidly depleted as myocardial fuels, whereas long-chain acylcarnitine levels significantly increased. This suggests that the graft may shift from fatty acid oxidation to other metabolic pathways during the early stages of perfusion [42]. Olkowicz et al. used GC/TOFMS to analyze the changes in metabolite levels in pig and human hearts during ESHP process. Pig heart metabolic processes involve multiple changes, including inflammation, oxidative stress, and disturbances in purine and fatty acid metabolism, whereas the human heart exhibits unique metabolic changes related to cardiolipin synthesis and mitochondrial membrane remodeling. These differences may be associated with the unique biological characteristics of the human heart [44].

Recently, temperature-controlled hypothermic preservation (TCHP) has demonstrated a reduced PGD compared to conventional standard cold storage (SCS) [45, 46]. Gaurav Sharma et al. demonstrated that TCHP maintains bioenergetic stability comparable to SCS using high-resolution ^1^H and ^31^P nuclear magnetic resonance (NMR) spectroscopy and LC–MS on frozen cardiac tissues. They observed similar levels of high-energy phosphates and energy charge ratios, indicating preserved bioenergetic stability in the TCHP system. In addition, TCHP exhibited higher lactate/alanine ratios in the left ventricle (3.76 ± 0.84 vs. SCS 2.08 ± 0.12, p = 0.0423) and left atrium (5.63 ± 0.29 vs. SCS 3.67 ± 0.62, p = 0.0438), with exclusive detection of β-hydroxybutyrate (β-HB), suggesting enhanced glycolytic and ketone metabolic flexibility in heart with TCHP [47].

#### Prediction of specific types of rejection: ACR or AMR

The gene expression profiling (GEP) monitoring system can analyze gene expression characteristics related to heart transplant rejection, enabling the non-invasive detection of specific types of CAR and potentially distinguishing ACR from AMR (Table 2). This multi-center prospective Cardiac Allograft Rejection Gene Expression Observational (CARGO) study is the first to use GEP to differentiate moderate-to-severe ACR (grade ≥ 3A) from non-rejection. The researchers used microarray technology to analyze peripheral blood mononuclear cells (PBMCs) from patients with ACR and confirmed 11 key genes from a set of 252 genes identified using alloimmune pathways and leukocyte microarrays. These genes were incorporated into an 11 gene quantitative real-time PCR (qRTPCR) assay, laying the foundation for the development of AlloMap [4]. AlloMap, a commercialized GEP product, is the first and only non-invasive analysis method recommended by the ISHLT guidelines for identifying ACR risk. The AlloMap test score results from the quantification of intracellular mRNA levels of 20 immune related genes and effectively identifies moderate-to-severe ACR (grade ≥ 3 A), guiding immunosuppressive therapy [52]. An Invasive Monitoring Attenuation through Gene Expression (IMAGE) study further validated the effectiveness of AlloMap. Patients were randomly allocated to either the AlloMap or EMBx group. With a threshold score of 34, both methods showed similar cumulative incidences of clinical adverse outcomes (hazard ratio [HR]: 1.04, 95% CI 0.67–1.68) [53]. GEP monitoring is not inferior to conventional EMBx in detecting ACR and can reduce the frequency of EMBx in low-risk patients 6 months after HTx [54]. Most patients were classified as having a low risk of rejection and did not require EMBx based on a score below 34. Although using a higher threshold may further reduce the frequency of required EMBx, the IMAGE results suggest that a score below 34 represents a prudent threshold for clinical practice, particularly for patients whose time post-transplant is > 6 months. The CARGO II study included a larger clinical cohort and used the same threshold of 34 to distinguish ACR (grade ≥ 3A/2R). The results showed that after 2–6 months of HTx, the average AUROC for AlloMap was 0.70 (95% CI 0.67–0.73) with an NPV of 98.4%. After 6 months of HTx, the average AUROC was 0.69 (95% CI 0.66–0.72) with an NPV of 98.3%, demonstrating good predictive capability for AlloMap across different time points after HTx [9]. Due to the lack of a strong PPV, AlloMap may be more suitable for low-risk patients between 6 months and 5 years after HTx to exclude the occurrence of 2R or higher-grade ACR [21]. In future, high-throughput sequencing technologies are expected to enable more comprehensive gene expression detection and further optimize the diagnostic capabilities of AlloMap.

**Table 2:** Application of transcriptomics in HTx

Study type	Rejection type	Technology	Recipients characteristics	Rejecting sample source	Validation	Experimental design	Conclusion	Ref
**Prediction of CAR and early graft injury**								
Multi-center Prospective study	CAR	qRTPCR, miR ISH	30 rejecting recipients, Mean age, y: 41 Male (%): 57	30 EMBx and PBMC, Time between HTx and sample collection, month: 22	Yes	qRTPCR and miR ISH were performed to test fourteen miRs expressed in EMBx and PBMC AUROC of miRs was performed to discriminate rejection and normal	MiR-10a, miR-21, miR-31, miR-92a, miR-142-3p miR-155, and miR-451 were differently expressed between normal and rejection allograft Four miRs highly discriminated rejection from normal: the AUROC of miR-10a: 0.975, miR-31: 0.932, miR-92a: 0.989, miR-155: 0.998	[62]
**Prediction of Specific Types of Rejection: ACR or AMR**								
ACR^†^								
Multi-center Prospective study (CARGO)	ACR	Microarray analysis, qRTPCR	28 rejecting recipients, Mean age, y: NA Male (%): 92.1	38 PBMC, Time between HTx and sample collection, month: 2.77	Yes	Microarrays and qRTPCR were performed to identify candidate genes LDA was used to covert gene expressions into scores and validated by AUROC	An 11 gene test distinguished the ACR (ISHLT grade ≥ 3A^††^) (p = 0.0018). At different scores of threshold, the AUROC for the ≥ 6 month and ≥ 1 year in validation cohort were 0.80 ± 0.14 and 0.86 ± 0.09, respectively Allomap was developed based on the study	[63]
Multi-center Randomized event-driven noninferiority trial (IMAGE)	ACR	Allomap	297 patients for GEP Allomap, Mean age, y: 53.9 Male (%): 82.2 305 patients for biopsy, Mean age, y: 54.3 Male (%): 81.6	297 PBMC, Time between HTx and sample collection, month: 13.0–36.0 (69.0%) 305 EMBx, Time between HTx and sample collection, month: 13.0–36.0 (68.2%)	No	A noninferiority comparison of Allomap and EMBx with respect to the composite primary outcome of rejection was performed	Patients who had received a cardiac transplant more than 6 months were monitored with Allomap at the threshold of 34 and EMBx. They had similar 2-year cumulative rates of the composite primary outcome: 14.5% and 15.3%, respectively. The corresponding hazard ratio was 1.04	[53]
Multi-center observational study (CARGOII study)	ACR	Allomap	41 rejecting recipients, Mean age, y: 50.8 Male (%): 79.0	Paired PBMC and EMBx Time between HTx and study enrollment, month: < 6 months (71.0%)	No	AUROC for the Allomap scores was performed with EMBx as reference for rejection status The study was performed to test for rejection surveillance of heart transplant recipients	Patients who had received a cardiac transplant more than 2 months were monitored with Allomap at the threshold of 34 and EMBx. The AUROC of the allomap was 0.70 The AUROC of the allomap was 0.69 for patients who had received a cardiac transplant more than 6 months	[9]
Single-center Prospective study	ACR	Nontargeted RNA-seq, qRTPCR	28 rejecting recipients, Mean age, y: 47.0 Male (%): 86.0	28 PBMC, Time between HTx and study enrollment, month: 5.3	Yes	RNA-seq was performed on the serum samples from recipients undergoing EMBx to identify potential marker, followed by AUROC of miRs tested by the means of qRTPCR in the validation cohort	MiR-144-3p was identified as best potential markers for detecting ACR AUROC for MiR-144-3p detecting rejection (Grade ≥ 3A/2R) was 0.801 (p < 0.0001), mild rejection (Grade 1R) was 0.631 (p < 0.01) in the validation cohort. MiR-144-3p was an independent predictor for the presence of ACR with odds ratio of 14.538 (p < 0.01)	[56]
Single-center Prospective study	ACR	qRTPCR	21 rejecting recipients, Mean age, y: 59.0 Male (%): 82.0	63 PBMC, Time between HTx and study enrollment, month: 1.9	Yes	qRTPCR was performed to examine the relative expression of 179 miRs, followed by AUROC of miRs tested in the validation cohort	AUROC of miR-181a-5p was 0.804 with sensitivity and specificity of 78% and 76%, respectively; and a negative predicted value of 98%	[55]
AMR								
Multi-center prospective cohort study	AMR	Microarray analysis	55 rejecting recipients, Mean age, y: 47.0 Male (%): 71.0	71 EMBx and PBMC, Time between HTx and sample collection, month: < 12 months (48.0%)	Yes	Microarray analysis was performed on the EMBx of patients with or without AMR to detect diagnostic transcripts AUROC of AMR related transcripts was performed in the validation cohort	The AMR selective gene sets accurately discriminated patients with AMR from those without AUROC of NK transcripts was 0.87, endothelial activation transcripts was 0.80, macrophage transcripts was 0.86, and interferon-γ transcripts was 0.84 (p < 0.0001)	[64]
Multi-center Prospective study	AMR	NanoString nCounter Microarray analysis	70 rejecting recipients	70 FFPE EMBx	Yes	NanoString nCounte Microarray analysis was performed on the FFPE EMBx to detect AMR-specific genes AUROC of these genes was performed in the validation cohort	AMR-specific genes concluded DARC, PECAM1, VWF AUROC of the genes set differentiating AMR from non-AMR was 79.88	[61]
**Distinguish ACR and AMR**								
miRs								
Basic experiment	CAR	microarray analyses	mouse	graft	No	Microarray analyses was performed on the transplants of mouse,followed by some experiments	The expression of miR-182 increases in mouse grafts during the rejection period and in GILs. miR-182 specifically binds to the 3'UTR of Foxo1 mRNA and inhibits Foxo1 protein translation	[65]
Basic experiment	ACR	qRTPCR, Microarray analysis	5 rejecting recipients, Mean age, y: 52.0 Male (%): 20.0	5 EMBx and PBMC, Time between HTx and sample collection, days: 14.0	No	qRTPCR and microarray analyses was performed, followed by some experiments	MiR-142-3p, miR-92a-3p, miR-339-3p, miR-21-5p were among the microRNAs enriched in the exosomes of heart transplant patients with ACR Exosomes released by effector T cells show increased miR-142-3p levels. After being transferred to endothelial cells, miR-142-3p binds to the 3'UTR of RAB11FIP2 mRNA, inhibiting RAB11FIP2 expression. This, in turn, enhances endothelial permeability and disrupts endothelial barrier function	[66]
Basic experiment	ACR	NanoString nCounter Microarray analysis, qRTPCR, ISH,WB	7 rejecting recipients, mouse	EMBx	No	NanoString nCounter Microarray analysis and microarray G4472B were performed to analyze miR and mRNA, followed by qRTPCR ISH and WB were performed to analyze downstream reaction	Both in human and mouse ACR processes, the expression of miR-21, miR-142-3p, miR-142-5p, miR-146a, miR-146b, miR-155, miR-222, miR-223, and miR-494 increased, while the expression of miR-149-5p decreased The target protein of miR-155, SPI1, which is implicated in IL-6 signaling, was found to be downregulated In a murine heart transplant model, genetic ablation or pharmacological inhibition of miR-155 attenuated ACR symptoms and prolonged graft survival	[14]
MMDx								
Multi-center prospective study	ACR AMR	MMDx	211 patients included rejection and normal patients	152 EMBx	No	MMDx was performed on a microarray platform to analyze mRNA expression from EMBx and it was compared with biopsy samples from kidney by PCA and prototype analysis When grouped by histological diagnosis or DSA status, the prototype groups and scores served as variables. AUROC was performed to test its diagnostic capacity	The distributions in PCA were similar between kidney and heart tissue AUROC estimates for EMBx samples were lower than for kidney samples. Each sample's highest. The AUROC for AMR was 0.81, for NR was 0.78, and for ACR was relatively low at 0.65 in EMBx samples	[67]
Retrospective study	ACR AMR	MMDx	135 patients included rejection and normal patients	228 EMBx included rejection and normal patients	No	MMDx, EMBxx with or without dd-cfDNA were analyzed for rejection. Paired samples were tested to validate the The clinical performance of MMDx	There was a degree of agreement between simultaneous use of MMDx, dd-cfDNA, and EMBxx. The consistency between MMDx and EMBxx was 84% (p < 0.001), and the consistency between MMDx and dd-cfDNA was 72% (p < 0.001)	[68]
**MiRs distinguish ACR and AMR**								
Multi-center Prospective study	ACR AMR	qRTPCR, miR ISH	30 rejecting recipients, Mean age, y: 41.0 Male (%): 17.0	30 EMBx and PBMC, Time between HTx and sample collection, month: 22.0	Yes	qRTPCR and ISH were performed to test fourteen miRs expressed in EMBx and PBMC AUROC of miRs was performed to discriminate rejection and normal	Four miRs were associated with ACR: the AUROC of miR-10a: 0.981, miR-31: 0.902., miR-92a: 0.977, miR-155: 0.984 Four miRs discriminated patients with AMR: the AUROC of miR-10a: 0.969, miR-31: 0.903., miR-92a: 0.984, miR-155: 0.986	[62]
Prospective study	ACR AMR	NGS sequence, qRTPCR	24 rejecting recipients	FFPE EMBx	No	The next-generation sequencing was performed on the EMBx. Logistic regression analysis was performed to create unique miR signatures as predictive models of each rejection. qRTPCR was carried out on the EMBx	11 MiRNAs were identified as biomarkers for rejection miR-27b-3p, miR-29b-3p, and miR-199a-3p could distinguish ACR, while miR-208a, miR-29b-3p, miR-135a-5p, and miR-144-3p were associated with AMR	[69]
Multi-center prospective study	ACR AMR	Small RNA Sequening	34 rejecting recipients	EMBx and PBMC	Yes	Small microRNA high-throughput sequencing was performed for differential gene expression analysis and regression models, A miR panel with high diagnostic accuracy was constructed and validated in both internal and external cohorts	12 miRs and 17 miRs were identified to discriminate ACR and AMR The AUROC of the miR panel to predict ACR was 0.92, The AUROC of AMR miR panel to predict ACR was 0.82	[70]
**MMDx Recognizes New Subtypes**								
Multi-center prospective study	NR-Minor	MMDx	645 patients included rejection and normal patients	489 EMBx	No	Microarray analyses was performed to detect rejection-associated RATs. AA analysis was performed to identify a new subgroup of rejection	A new subgroup of rejection, NR-Minor was detected which exhibited mild inflammation and a higher rate of DSA positivity Genes in NR-Minor was associated with AMR and/or IFNG-induced chemokines such as CXCL9, CXCL11, NK chemokine CCL4, seven major class II HLA genes, and IFNG-induced genes like GBP1, GBP4, and GBP5	[71]

*CAR* cardiac allograft rejection, *qRTPCR* quantitative real-time polymerase chain reaction, *miR* ISH microRNA in situ hybridization, *y* year, *EMBx* endomyocardial biopsy, *PBMC* peripheral blood mononuclear cell, *HTx* heart transplantation, *miR* microRNA, *AUROC* area under the receiver operating characteristic curve, *miR* microRNA, *ACR* acute cell-mediated rejection, *CARGO* Cardiac Allograft Rejection Gene Expression Observational study, *LDA* linear discriminant analysis, *GEP* gene expression profiling, *IMAGE* Invasive Monitoring Attenuation through Gene Expression, *NA* not available, *Cir miR* circulating microRNA, *RNA-seq* RNA sequencing, *LncRNA* long non-coding RNA,*ncRNA-seq* non-coding RNA sequencing, *AMR* antibody mediated rejection, *NK* natural killer, *FFPE* formalin-fixed paraffin-EMBx edded, *DARC* duffy antigen receptor for chemokines, *PECAM1* platelet endothelial cell adhesion molecule 1, *VWF* von willebrand factor, *GILs* graft-infiltrating lymphocytes, *3'UTR* 3'untranslated region, *Foxo1* forkhead box O1, *RAB11FIP2* RAB11 family interacting protein 2, *MMDx* Molecular Microscope Diagnostic System, *DSA* donor-specific antibodies, *NR* no rejection, *PCA* principal component analysis, *dd-cfDNA* donor-derived cell-free DNA, *NGS* next generation sequencing, *RATs* rejection-associated transcripts, *AA* archetypal analysis, *CXCL9* C-X-C motif chemokine ligand 9, *CXCL11* C-X-C motif chemokine ligand 11, *CCL4* C–C motif chemokine ligand 4, *HLA* human leukocyte antigen, *IFNG* interferon gamma, *GBP1* guanylate binding protein 1, *GBP4* guanylate binding protein 4, *GBP5* guanylate binding protein 5

^†^AlloMap (CareDx Inc., Brisbane, CA), a noninvasive gene expression profiling assay, was utilized for the surveillance of acute cellular rejection

^††^The grading of ACR followed the ISHLT (the International Society for Heart and Lung Transplantation) standardized criteria

Therefore, the early diagnosis of different grades of ACR is necessary. Several independent studies have demonstrated the predictive value of circulating serum microRNAs (miRs) in serum. qRTPCR analysis of serum samples from 21 HTx patients revealed that miR-181a-5p had an AUROC of 0.804 (95% CI 0.707–0.880) when identifying ACR (grade ≥ 3A/2R), with a sensitivity of 78% and specificity of 76% [55]. In another study, RNA-seq analysis of consecutive serum samples from 40 HTx patients identified circulating miR-144-3p as the only predictor with an AUROC greater than 0.95 in ACR (grade ≥ 2R). Further validation in a cohort of 212 patients showed an AUROC of 0.801, with an OR of 14.538 (95% CI 2.348–90.02, p < 0.01) in distinguishing ACR (grade ≥ 1R), and an AUROC of 0.631 [56]. These findings suggest that ACR is associated with differential expression of specific serum miRs, which correlates with the severity of the rejection episode.

Moreover, advances in proteomic technologies have provided significant opportunities to predict different levels of ACR [57]. Isotope labeling (tandem mass tag, TMTs 10-plex) LC–MS analysis is a non-immunological, non-targeted, and unbiased method that can discover rejection biomarkers [58]. Based on the same isotopes chemical labeling in chromatography, which results in comigration phenomena, provides more accurate quantification with high specificity, sensitivity, and throughput, making it more accurate and reliable than traditional immunoassays. Plasma samples from transplant recipients were analyzed and 17 differentially expressed proteins were identified as potential ACR biomarkers. Among these, CD5L, a key inhibitor of macrophage apoptosis, showed the best results (AUROC: 0.850 ± 0.07, p < 0.01), being the only protein biomarker with an AUROC greater than 0.90 for detecting ACR (grade ≥ 2R). It also maintained good detection ability when comparing patients with no rejection or mild ACR (grade = 1R) (AUROC: 0.790 ± 0.120, p < 0.05). A multivariate logistic regression model further confirmed that CD5L was the only independent predictor of ACR (OR: 14.74, p < 0.0001). Owing to the heterogeneity in baseline patient characteristics, treatment, and clinical features, it may be necessary to use a combination of proteins rather than a single biomarker to improve the sensitivity, specificity, PPV, and NPV for detecting ACR. Incorporating CD5L as a candidate molecule in molecular combination panel assays may help improve the diagnosis and prevention of ACR [58].

Clinically, there are limitations in using immunopathological monitoring for AMR, with only approximately 60% of patients having biopsy samples that can confirm AMR via donor-specific antibodies (DSA) [59]. The American Heart Association emphasizes that an important gap remains in understanding the pathophysiology of AMR and improving the detection of activity, degree of injury, and stage [60]. A French multi-center prospective study used microarray technology to analyze 240 EMBx samples, demonstrating extensive molecular phenotyping of AMR for the first time. The AMR selective gene sets accurately discriminated AMR patients from non-rejection patients and included natural killer (NK) cell transcripts (AUROC: 0.87), endothelial activation transcripts (AUROC: 0.80), macrophage transcripts (AUROC: 0.86), and interferon (IFN) -γ transcripts (AUROC: 0.84) [53]. The nCounter (NanoString) microarray technology directly counts molecular tags using fluorescently labeled probes, avoids amplification steps and offers high sensitivity and quantitative accuracy, making it suitable for detecting formalin-fixed paraffin-embedded (FFPE) samples. Analysis of FFPE EMBx samples from patients with AMR showed that the expression of the preselected AMR gene sets was significantly higher than that in normal samples (p < 0.0001). In terms of AMR detection efficacy, gene set expression showed higher accuracy than DSA (AUROC: 70.47 vs. AUROC: 79.88) [61]. However, the gene screening range of microarray technology is limited. Future genome-wide association studies may expand the screening scope and deepen the understanding of the pathological mechanisms of AMR, with prospective validation in larger cohorts.

### Unveiling the mechanisms of rejection and distinguishing ACR from AMR

Because the initial treatment strategies for the two types of CAR differ markedly and AMR is associated with more severe graft dysfunction, higher recurrence rates, long-term graft damage, and increased mortality, accurately differentiating between rejection types has important clinical value. The ISHLT emphasizes the need for individualized immunosuppressive therapy based on a patient's rejection risk [72]. A deeper understanding of the pathophysiological differences between ACR and AMR could help distinguish between the two types of rejection. AMR is primarily associated with the production of circulating DSA in patients, which activates immune cells and the complement systems, and causes graft damage. This leads to diffuse large vessel injury and progression to microcirculatory inflammatory damage [10, 59]. GEP analysis suggests that IFN-γ effects exacerbate tissue damage in AMR, where IFN-γ upregulates chemokines such as CX3CL1, CXCL10, and CXCL11, promoting immune cell recruitment, enhancing microcirculatory inflammation, and increasing myeloid cell FCER1G expression and CSF2RB activity, thus increasing cytotoxicity and inflammation [64]. The expression of IFN genes should be prospectively evaluated to determine their potential as early diagnostic markers and therapeutic targets in AMR.

Non-human leukocyte antigen (HLA) antibodies may also contribute to the development and progression of AMR through their potential mechanisms of action related to antigen function [73]. The affinity chromatography shotgun immunoproteomics approach can identify multiple non-HLA targets and their longitudinal changes, overcoming the limitations of previous studies that only focused on a single antigen [13, 74]. During transplantation and acute rejection episodes, specific immunoglobulin G (IgG) antibodies targeting non-HLA antigens are used to create affinity columns. These columns can capture the corresponding antigens from donor heart biopsy samples collected at the time of transplantation, allowing for subsequent quantitative proteomics analysis. Among the 155 non-HLAs in the DSA-negative AMR patients, 43 high-prevalence antigens were found in approximately 50% of the patients. Researchers identified four high-prevalence antigens related to myocardial or endothelial cell dysfunction: JUP, TT, PHB, and ATPS. In vitro experiments showed that non-HLA antibodies could induce myocardial dysfunction and endothelial cell proliferation, further linking these antigen–antibody reactions to changes in myocardial and endothelial cell function. Considering the presence of anti-JUP antibodies during acute AMR and increased antigen abundance in the graft, JUP may be the primary initiator of AMR. In contrast, TT, PHB, and ATPS are intracellular antigens, and their antibody levels increase significantly during the chronic rejection phase, possibly serving as secondary antigens that enter the immune system after the initial graft injury or acute rejection. Therefore, it is necessary to use single antigen-specific screening assays to assess larger patient cohorts and validate the predictive value of each non-HLA antigen for AMR [13].

In contrast, ACR is associated with more localized lymphocytic infiltration and injury, often confined to the interstitium and perivascular tissue spaces [10, 18]. CD5L is a soluble protein of approximately 40 kDa that is primarily produced in inflamed tissues and directly inhibits macrophage apoptosis. The role of this macrophage apoptosis inhibitor in cardiovascular diseases has been well established, particularly in atherosclerosis and myocardial infarction, where CD5L deficiency has been shown to attenuate inflammation and reduce infarct size, thereby improving survival outcomes [75, 76]. Several proteomic studies have identified the important role of CD5L in regulating inflammation, suggesting that the regulation of macrophage apoptosis is directly involved in the pathophysiology of ACR [58, 77]. Notably, AMR is associated with the accumulation of macrophages in blood vessels, and CD5L is a potential biomarker for AMR, which warrants further investigation.

MiRs serve as key regulators of CAR, delineating the distinct developmental trajectories of various CAR subtypes and providing a theoretical basis for potential therapeutic strategies. The miR expression profile in a mouse heart transplant rejection model showed that miR-182 is significantly increased in both PBMCs and plasma during graft rejection. MiR-182 inhibits Foxo1 protein translation by specifically binding to the 3'untranslated region (UTR) of Foxo1 mRNA [65]. Another study found that four miRs (miR-10a, miR-31, miR-92a, and miR-155) exhibited differential serological expression with a strong correlation with their tissue expression (p < 0.0001) [62]. MiR-10a has been shown to inhibit the NFkB signaling pathway, consequently increasing the pro-inflammatory markers MCP-1, interleukin (IL) −6, IL-8, IL-1, and VCAM in endothelial cells [78]. MiR-155 is upregulated after T-cell receptor activation, suppresses IFN receptor expression, and participates in B cell immunoglobulin switching, thereby regulating immune responses [79]. MiR-31 regulates the expression of E-selectin and ICAM-1 through the tumor necrosis factor (TNF) pathway, influencing integrin expression and immune cell infiltration [80]. MiR-92a targets integrin α5, S1P1, MKK4, and eNOS, showed endothelial cell chemotaxis and potentially played a role in vascular inflammatory responses [81]. Van Aelst et al. found that miR-155 was significantly upregulated in patients with ACR and affected the IL-6 signaling pathway by downregulating SPI1 protein levels, exacerbating cardiac inflammation. In an ectopic heart transplant mouse model, deletion and pharmacological inhibition of miR-155 attenuated the ACR reaction, suggesting a non-organ specific signature of acute rejection [14].

However, research exploring the pathophysiological mechanisms of miRs and their potential as biomarkers is limited by data conflicts and individual patient differences, resulting in large datasets that increase workload while also having a high rate of false positives and false negatives [62]. Exosomes are cell-derived circulating vesicles that assemble and carry mRNA, miRs, and proteins that are easily detectable in the circulation and contain a relatively limited set of bioactive molecules [82]. Identifying changes in the exosomal protein content in the serum of patients with rejection may reflect the pathogenic mechanisms of ACR and AMR. Dewi et al. studied the interaction between miRs and the allogeneic heart transplant endothelium in ACR. They found that the miR-142-3p released by effector T cells was increased in the serum of patients with ACR. After being transferred to endothelial cells, miR-142-3p bound to the 3'UTR of RAB11FIP2 mRNA, inhibiting its expression and enhancing endothelial permeability, thereby disrupting endothelial barrier function [66]. Kennel et al. used liquid chromatography-tandem mass spectrometry (LC–MS/MS) to identify 15 proteins that were differentially expressed in ACR, AMR, and non-CAR samples. Most of these proteins are related to complement activation, immunoglobulins, and coagulation, and are linked to adaptive immunity [83]. Except for certain immunoglobulin subcomponents, the levels of differentially expressed proteins were reduced in both ACR and AMR samples compared to non-CAR HTx samples. This may reflect the depletion of serum exosomes enriched in immune and hemostatic mediators, which are increasingly utilized by host cells during rejection responses. AMR is associated with increased complement deposition in capillary microthrombi and allogeneic transplant microvascular disease.

Currently, available technologies and miRs are promising for differentiating between ACR and AMR. In a real-world retrospective study, heart molecular microscope diagnostic (MMDx) analyzed RNA transcripts from transplanted heart tissue to differentiate ACR, AMR, injured, and healthy tissue, overcoming the limitations of traditional microarrays that only detect one rejection type [68]. The study included 135 adult HTx patients with 228 corresponding EMBx and MMDx specimens, along with dd-cfDNA. The diagnostic consistency between MMDx and EMBx was 84% (p < 0.001) and that with dd-cfDNA was 72% (p < 0.001). Among the 37 discordant samples, MMDx classified 32 as rejected, whereas EMBx detected only 5. Three patients received treatment earlier owing to MMDx-positive results and benefited clinically. Halloran et al. used MMDx to analyze rejection-associated transcripts in 331 HTx patients, providing a new dimension for improving ACR and AMR diagnostic accuracy. The results showed that the AUROC for AMR was 0.81, and for ACR, it was lower at 0.65, possibly because of pathologists'lower consistency in EMBx histological diagnoses [67]. MiRs also have potential as biomarkers for distinguishing ACR from AMR. To distinguish ACR, the AUROCs were 0.981 for miR-10a, 0.902 for miR-31, 0.977 for miR-92a, and 0.984 for miR-155. For AMR, the AUROCs were 0.969 for miR-10a, 0.903 for miR-31, 0.984 for miR-92a, and 0.986 for miR-155 (p < 0.0001) [62].

NGS significantly enhanced the identification of specific miRs associated with the CAR. Eleven miRNAs were identified as potential biomarkers of rejection in the EMBx samples from 33 HTx recipients. Among these, miR-27b-3p, miR-29b-3p, and miR-199a-3p were associated with ACR, whereas miR-208a, miR-29b-3p, miR-135a-5p, and miR-144-3p were associated with AMR [69]. The GRAft study employed high-throughput small RNA sequencing to analyze plasma samples from HTx recipients who experienced rejection. Differential gene expression and regression analyses identified 12 miRs specific to ACR and 17 miRs specific to AMR. A diagnostic miRs panel was developed and validated in clinical cohorts. The AUROC was 0.92 (95% CI 0.86–0.98) for ACR and 0.82 (95% CI 0.74–0.90) for AMR [70]. Additionally, an miR-based scoring system was established to quantify the likelihood of ACR and AMR on a scale of 0–100. Establishing a threshold score of 65, the diagnostic performance for ACR yielded an AUROC of 0.86 (95% CI 0.79–0.93), with 86% sensitivity, 76% specificity, and a NPV of 98%. For AMR, the AUROC was 0.84 (95% CI 0.78–0.91), with 82% sensitivity, 84% specificity, an NPV of 97%, and a PPV of 37%.

### Omics application in CAV

Novel biomarkers may address the insufficiency of the ICA and IVUS for the early diagnosis of CAV and have a substantial impact on CAV intervention [7]. Holzhauser et al. leveraged AlloSure first showed that significant CAV (grade ≥ 1) was more likely to be present in patients with elevated dd-cfDNA levels (≥ 0.12%) compared to those with low dd-cfDNA levels (< 0.12%) [84]. Nevertheless, dd-cfDNA may not be an effective diagnostic method to replace ICA. Jimenez-Blanco et al. used the ICA as the gold standard to prospectively observe the %dd-cfDNA quantified using NGS. A comparison of dd-cfDNA levels in patients with CAV0 and different grades of CAV did not show significant differences (0.92% vs. 0.46%, p = 0.059), or between patients with stable CAV (no new coronary lesions since the previous angiogram) and those with progressive CAV (0.7% vs. 0.9%, p = 0.76) [49]. Another AlloSure study supported this point, the median dd-cfDNA level was not significantly different between CAV (−) and CAV (+) samples (0.09% vs. 0.15%, p = 0.25), and similar results across all CAV grades (CAV0 = 0.09%, CAV1 = 0.17%, CAV2 = 0.17%, CAV3 = 0.13%). These results indicate that %dd-cfDNA was not associated with the presence or severity of CAV [6]. Nonetheless, elevated dd-cfDNA levels may indicate graft ischemia, and patients with severe CAV and graft dysfunction are susceptible to ischemia and graft injury. Therefore, it is necessary to determine the role of dd-cfDNA in patients at a high risk of severe CAV.

Compared to dd-cfDNA, GEP may reveal a potential pathological mechanism and identify patients with early CAV risk. Microarray integration with ICA effectively distinguished patients with CAVs. Differential gene pathway analysis significantly enriched in macrophage activation, interleukin-6 pathway, NF-κB cascade and response to virus [85]. A cross-sectional study using targeted qRTPCR showed the diagnostic value of circulating levels of endothelium-enriched miRs in CAV. Median plasma levels of miR-210-5p, miR-92a-3p, miR-126-3p, and miR-126-5p were higher in patients with CAV than in those without. Among them, miR-92a-3p and miR-126-5p exhibited excellent CAV discrimination capabilities (C statistic: 0.682, 95% CI 0.533–0.831; C statistic: 0.655, 95% CI 0.502–0.807) [86]. Neumann et al. confirmed that circulating miR-628-5p was a potential biomarker of severe CAV (AUROC: 0.808). When miR-628-5p was > 1.336 for predicting CAV, the sensitivity was 72.22% and the specificity was 83.33%, providing a feasible approach for early identification and personalized intervention in patients at high risk of severe CAV [87].

CAV-specific proteins may explain the differences in pathogenesis between early mild-to-moderate and late severe CAV and hold potential therapeutic value. The Slow Off-rate Modified Aptamer (SOMAscan) assay, an efficient and repeatable proteomics biomarker discovery tool that can analyze over 1000 protein targets simultaneously in only 150 μL of serum based on aptamers [72, 88]. Almufleh et al. retrospectively used SOMAscan to evaluate the serum from 21 patients with different CAV grades, and identified ICAM-2, PPID, SPOCK1, and N6AMT1 as specific markers for mild-to-moderate CAV. The function of N6AMT1 and its association with CAV pathogenesis remain unclear. Other proteins have been confirmed to be involved in regulating apoptosis and ischemia–reperfusion injury, which may be related to the early stages of CAV injury. In cell injury and apoptosis pathways, RPS7 was increased in patients with CAV and has been previously demonstrated in ovarian tumor cell lines to have pro-apoptotic functions mediated via MAPK and mammalian target of rapamycin (mTOR) signaling pathway [89]. HIST2H2BE and PRKACA were also upregulated in CAV and are involved in regulating fibrosis and apoptosis [90]. The elevation of inflammatory markers such as C-reactive protein, TNF-a, IL-1B and uric acid suggested inflammatory infiltration during the development of CAV. Conversely, ERBB3, a peripheral mononuclear cell receptor enzyme and a regulator of inflammation, significantly reduced levels in CAV. Lower ERBB3 levels and increased pro-inflammatory cytokines levels lead to poor prognosis for CAV. These candidate biomarkers were validated in prospective studies to predict CAV ability and potential targets, and the preliminary AUROC curve had shown satisfactory ability to distinguish CAV (AUROC: 0.72–0.94) [88]. In addition, Nevarez-Mejia et al. employed GeoMx digital spatial profiling (DSP) to conduct patho-molecular and spatial analyses of CAV + DSA + rejected cardiac allografts [91]. DSP revealed the proteomics and transcriptomics characteristics of the CAV regulatory pathway, including elevated levels of transforming growth factor (TGF) -β-regulated transcripts and platelet activation/aggregation modules, correlating with growth factor-associated proteins and proliferation or repair pathways.

Other potential biomarkers include clonal genesis of indeterminate potential (CHIP), a phenomenon in which hematopoietic stem cells acquire somatic mutations [92]. Mutations in DNMT3A, ASXL1, and TET2 are the main risk factors for cardiovascular disease and death in patients after HTx. In different studies, the ability of CHIP to predict long-term graft injury in patients after HTx has been contradictory. CHIP mutations may not be related with the risk of CAV or increased post-transplant mortality [48, 50]. Nevertheless, it is essential to prospectively evaluate CHIP at different sequencing time points to determine its potential as a therapeutic target for augmenting the post-transplant clinical course.

### Individual monitoring and promotion of omics clinical transformation

Several factors affect the clinical management of HTx dd-cfDNA through non-invasive monitoring. The sample preparation method affects the accuracy of dd-cfDNA detection. Peripheral blood cell lysis may dilute the dd-cfDNA signal and hinder the differential recognition of rejection in HTx patients. The DNA-Based Transplant Rejection Test (DTRT) compared the whole blood-protocol with the plasma-protocol, which centrifuged blood samples to quickly separate the plasma, immediately identified leukocyte dissolution, and removed samples that exceeded a conservative threshold level. These results indicate that the dd-cfDNA detection performance of the plasma-protocol was superior to that of the whole blood-protocol at different thresholds [93]. The median %dd-cfDNA levels of samples collected retrospectively and prospectively were similar (0.06% vs. 0.04%), and the prevalence of CAR in the retrospective samples was higher than that in the prospective samples (12.0% vs. 5.1%). However, differences in baseline conditions might reduce reliability [27].

Most dd-cfDNA studies have only included adult patients; however, dd-cfDNA assessment is also an effective alternative for invasive detection in pediatric HTx. Verhoeven et al. utilized ddPCR to simultaneously detect %dd-cfDNA (r = − 0.02, p = 0.74) and dd-cfDNA absolute levels (r = −0.04, p = 0.64) in HTx adults and children, and showed an insignificant correlation between age and fold change [32]. Therefore, dd-cfDNA assessment may be more practical for pediatric patients with low immunity and invasive surgical tolerance. It is also useful for optimizing immunosuppressive regimens for pediatric transplantation [12, 93].

The results of shotgun sequencing of female and male in a multi-center prospective study identified that the median level of %dd-cfDNA between ACR and AMR in gender was non-significant (0.33% vs. 0.32%, p = 0.57; 0.50% vs. 0.63%, p = 0.51), and similar detection performance characteristics (AUROC: 0.83, 95% CI 0.75–0.91 vs. AUROC: 0.89, 95% CI 0.85–0.94) [19]. These findings suggest that similar diagnostic thresholds can be implemented for rejection monitoring in different sexes. For diverse races, in the shotgun sequencing results of GRAfT, the incidence of CAR was higher in Black patients compared with White patients (43% vs. 19%, p = 0.002). AMR occurred predominantly in Black patients with a prevalence of 20% versus 2% (p < 0.001), and the primary composite outcome was higher in Black patients than in White patients (p < 0.001) [25]. This might have led to higher % dd-cfDNA levels in black patients than in White patients (0.09% vs. 0.05%, p = 0.003) after transplantation, which continued to increase. Consequently, improving our understanding of the differences in transplantation prognosis among different races may facilitate the establishment of personalized clinical care and immunotherapy. Regrettably, there is a lack of comparative dd-cfDNA data between Yellow patients and others.

GEP, a non-invasive monitoring method, provides a reliable alternative for ACR diagnosis at different stages in patients with HTx. Extended Molecular Immunology for Allograft Gene Expression (EMIAGE) confirmed GEP as an alternative to EMBx or the gold standard for detecting moderate-to-severe ACR. The GEP thresholds were set to 30 and 34 for patients at 2–6 months and > 6 months after HTx, respectively. The primary endpoint included a composite of death or retransplant, rejection with hemodynamic compromise, or graft dysfunction at 18 months after transplantation. The results indicate that the incidence of the primary endpoint was similar between the GEP and EMBx groups (10% vs. 17%, p = 0.44) [94]. Actually, the clinical transformation of GEP also requires consideration of a variety of factors. Clinically, the appropriate AlloMap threshold was determined according to the condition of the HTx patients to effectively exclude ACR. CARGO and CARGOII revealed that the steroid dose continued to decrease within the first year after HTx, and the average GEP score showed an upward trend at 2–6 months, tended steadily, and stabilized after 1 year. Therefore, for patients with a high risk of ACR in the early stage (the first 6 months after HTx), low thresholds may be more suitable for the timely detection of rejection [9, 63]. Although whether the threshold should be adjusted according to age remains inconclusive, the CARGO II multivariate model noted that the risk of ACR was related to age and that the risk of ACR (grade ≥ 3A/2R) was significantly reduced in patients aged ≥ 54 years (p = 0.008) [9].

AlloMap is limited to detecte only ACR, but exosome proteomics can simultaneously detect ACR and AMR compared with AlloMap, thus affording a safe, non-invasive, and effective alternative to EMBx for the diagnosis of rejection.

### Innovative targeting strategies provided by single-cell technologies

Single-cell omics technologies include single-cell RNA sequencing (scRNA-seq) and single-cell T cell receptor (TCR) sequencing (scTCR-seq). Importantly, they can be leveraged to characterize dynamic cell-specific transcriptomics profiles in different disease states and identify novel biomarkers and disease-relevant pathways to achieve targeted therapy [15].

Immune cell populations in grafts and their associated transcriptomics and metabolic activities are complex. To data, multiple studies have demonstrated that scRNA-seq accurately resolves the intercellular signaling networks of allogeneic immune responses, identifying novel cell subtypes and intercellular communications associated with CAR [95–97]. Kong et al. combined scRNA-seq and scTCR-seq to elucidate the dynamic landscapes of cardiac fibroblasts (CFBs) and T cells in mouse allogeneic heterotopic HTx, as well as the potential pathogenesis and therapeutic targets of ACR [95]. In the ACR group, the proportion of CFBs decreased sharply. However, highly expanded cytotoxic T lymphocytes (CTLs) and a CXCL10^+^Gbp2^+^ subcluster of CFBs were enriched within the grafts at the late stage. The latter features strong IFN responsiveness and high expression of chemokines and major histocompatibility complex molecules (MHC), which participate in the recruitment and activation of immune cells. Cell–cell communication analysis suggested that the CXCL9/CXCL10-CXCR3 axis might contribute to the regulation of chemotaxis and immune cell recruitment, and that targeted inhibition of CXCR3 increased graft survival. In addition, CFBs interact strongly with myeloid and T cells, and intervention in the CXCL9/CXCL10-CXCR3 axis could reduce CD4^+^ T cell infiltration and prevent adverse cardiac remodeling [98]. A recent study clarified that the ACR immune atlas in mouse HTx supported the CXCR3 axis as a potential therapeutic target [99]. Cell–cell communication and flow cytometry confirmed that CXCR3 and its ligands were upregulated in ACR. It is noteworthy that most studies have targeted only one molecule (receptor or ligand) within the CXCR3 signaling pathway, thus failing to completely block this pathway. Chen et al. combined the CXCR3-173 mAb with the MIG-2F5.5 mAb to completely block the CXCR3 pathway, which was more effective than a single neutralizing antibody in inhibiting alloreactive T cell function [99]. These findings provide innovative insights for rejection therapy and drug development. Future studies will require fully blocking antibodies targeting the CXCR3 pathway.

Damage associated molecular patterns (DAMPs) are recognized through pattern recognition receptors (PRRs) on the cell surface and in the cytoplasm of innate immune cells. This process activates innate immune cells and assists in the occurrence of the CAR [100]. In HTx, inhibition of MHC-I antigen-specific memory recognition of monocytes and macrophages can reduce rejection [101]. Therefore, the development of immunosuppressive agents targeting the innate immune system warrants further investigation. scRNA-seq significantly enhanced the ability to identify innate immune cell populations in the CAR. Monocytes and macrophages are actively involved in antigen presentation, inflammatory cell recruitment, and glycolysis. HIF1A transcriptional regulation promoted the increase of glycolysis, while the key rate-limiting enzyme of glycolysis, PKM2, promoted the increase of *Hif1α* expression in macrophages under normoxic state, forming a positive feedback loop. Both the mouse CAR model and human CAR specimens confirmed that glycolysis and HIF1A were increased in macrophages, which provided a theoretical basis for targeted inhibition of HIF1A to reduce CAR [102]. After HIF1A inhibition, the antigen-presenting ability, pro-inflammatory ability, and T cell infiltration of allograft macrophages were significantly reduced, indicating that HIF1A may affect adaptive immunity by regulating the innate immune response in CAR. Additionally, upregulation of IFN activation and pro-inflammatory polarization genes expression were observed in mouse allografts innate immune cell infiltration. NK cells, the main source of IFN-γ signaling, activating inflammatory monocytes, showing pronounced MHC and co-stimulatory molecular signals. In inflammatory monocytes, caspase-1 exhibited specific upregulation in response to IFN signaling. In *vivo*, inhibition of caspase-1 decreased immune infiltration, prevented CAR, and improved cardiac systolic function [103]. Small-omics studies assessing targeted proteomics and serum levels of miRs in CAV have been proposed as possible strategies for the early diagnosis of CAV but show limited sensitivity and specificity [86, 104, 105]. The main therapy, mTOR inhibitors, focused on interrupting CAV pathogenesis following diagnosis, is primarily limited by the adverse effects of drugs [106, 107]. To develop novel therapies to prevent the occurrence and development of CAV and prolong the survival of allografts, it is necessary to perform scRNA-seq in CAV. Recent studies have shown that immune and non-immune cells strongly express inflammation and angiogenesis-associated genes in CAV patients [108]. Compared to low-grade CAV (CAV0 and CAV1), CD4^+^ T central memory cells, CD14^+^ and CD16^+^ monocytes were significantly increased in high-grade CAV (CAV2 or CAV3) and were enriched in leukocyte differentiation, angiogenesis and inflammatory response pathways. Furthermore, the intersection with the druggable genome prioritized 68 targets, including IFN and IFN response genes (IFNG, IFIT1, ISG15, IL1A, IL1B, IL1RAP, ITGB7, etc.) [108]. Notably, partial genes expression precedes CAV development and predicts disease progression, thereby identifying patients who may benefit from mTOR inhibitor therapy. In contrast, other target genes, as manifestations of the response to CAV, provide innovative ways to inhibit the CAV pathogenesis.

### Omics combined with machine learning

Obtaining extensive omics data from large-scale cohorts increases the complexity and challenges of the analysis. Machine learning, an efficient and convenient strategy for solving problems related to data processing and analysis. Researchers have built various prediction models through internal or external verification and cross-validation to improve the biomarkers identification accuracy [114, 115]. Presently, machine learning has been widely employed in HTx proteomic data analysis (Table 3).

**Table 3:** Proteomics combined with machine learning

Author	Technology	Machine learning type	Sample type	Patients cohort	Proteins finding	Main finding	Ref
Yuling Yu et al. (2024)	CE-MS	SVM; Logistic regression model	Urine	Number of patients^†^ n = 389; training cohort n = 140; validation cohort n = 118	Number of proteins: 1576 peptides; Protein biomarkers: OSTEO18	OSTEO18, containing 18 urine proteomic biomarker peptides, predicting osteoporosis in HTx receptors and is currently confirmed in further in *vitro* diagnosis	[109]
Dongmei Wei et al. (2022)	CE-MS	Decision tree; Logistic regression model	Urine	Number of patients^††^ n = 217; training cohort^†††^ n = 108; validation cohort^†††^ n = 109	Protein biomarkers^*^: 27 peptides construct classifier	Identifying and validating 27 peptide urinary proteomic features for monitoring CAV. Providing insights into the pathological process of CAV, including collagen biosynthesis and degradation, platelet aggregation and coagulation, cell-extracellular matrix interaction, cell adhesion and motility	[104]
Nicholas P. Giangreco et al. (2022)	LC–MS	Logistic regression model; MCCV	Serum	Number of patients n = 88	Number of proteins: 681; Protein biomarkers: F2, a2-SERPINF2, F9, CPB2, HGFAC, LK	The EVs proteome SERPINF2, F9 and LK could predict survival, AUROC > 0.6. Survival after HTx was associated with platelet activation and shift in the kallikrein kinin system away from vasodilation and towards coagulation	[110]
Nicholas P Giangreco et al. (2021)	LC–MS	Logistic regression model; MCCV	Serum	Number of patients n = 88	Number of proteins: 681; Protein biomarkers: KLKB1, PRDX2, TPM4, MPO	The level of KLKB1 before HTx was a reliable predictor of PGD after HTx. The proteomic features of PGD predicted before HTx were enriched in inflammatory and immune pathways	[111]
Lauren K. Truby et al. (2021)	Olink, PEA	Logistic regression model	Serum	Number of patients n = 219; derivation cohort^**^ n = 131; Validation cohort^***^ n = 88	Number of proteins: 354; Protein biomarkers: CLEC4C	The level of circulating CLEC4C before HTx distinguished the risk of PGD in patients with HTx. Integrated with clinical covariates in multivariate models enabled to improve the clinical main results	[112]
Chiara Castellani et al. (2020)	Nanoparticle tracking analysis; Multiplex flow cytometry assay	RF	Plasma	Number of patients n = 90; training cohort n = 53; validation cohort n = 37	Number of proteins: 37; Protein biomarkers:CD3, CD2, ROR1, SSEA-4, HLA-I, CD41b, HLA-II, CD326, CD19, CD20, CD25	The integration of plasma-derived EVs-specific antigen markers and HTx patients management using machine learning models promoted clinicians determination. EVs-markers were enriched in immune system and signal transduction-related pathways, involving inflammatory responses, intercellular communication, cell survival, and apoptosis	[113]

*HTx* heart transplantation, *CE-MS* capillary electrophoresis coupled with mass spectrometry, *SVM* support vector modeling, *CAV* cardiac allograft vasculopathy, *LC–MS* liquid chromatograph mass spectrometer, *F2* prothrombin, *a2-SERPINF2* alpha 2-antiplasmin, *F9* coagulation factor IX, *CPB2* carboxypeptidase 2, *HGFAC* hepatocyte growth factor activator, *LK* low molecular weight kininogen, *MCCV* monte carlo cross validation, *EVs* extracellular vesicles, *PGD* primary graft dysfunction, *KLKB1* plasma kallikrein, *PRDX2* peroxeridoxin 2, *TPM4* tropomyosin alpha-4, *MPO* myeloperoxidase, *PEA* proximity extension assay, *CLEC4C* C-type lectin domain family 4 member C, *WB* western blotting, *RF* random forest, *SSEA-4* non-immune system-related antigens, *CD41b* the platelet membrane glycoprotein II-b, *HLA* human leukocyte antigen, *CD326* the epithelial cell adhesion molecule, *CD20* B-lymphocyte antigens, *CD25* the interleukin-2 receptor alpha chain, *EMBx* endomyocardial biopsy

^†^154 Osteoporotic heart transplant patients

^††^76 patients with CAV

^†††^38 patients with CAV

^*^22 peptides annotating to 7 different proteins and 5 unknown peptides, including collagen I, collagen II alpha I chain, collagen III alpha I chain, collagen XI alpha 1 chain, mucin-1 subunit alpha, xylosyltransferase 1, and protocadherin-12

^**^39 PGD, 24 moderate and 15 severe PGD

^***^26 PGD, 18 moderate and 8 severe PGD

In HTx patients management, machine learning efficiently assists clinicians to determine whether EMBx is required. Castellani et al. evaluated the concentration of extracellular vesicles (EVs) in plasma using nanoparticle tracking analysis (NTA) and reported that the EVs concentration was significantly increased in patients with CAR (p < 0.001). Among the EVs surface markers, CD3, CD2, ROR1, SSEA-4, HLA-I and CD41b were identified as discriminants between controls and patients with ACR, whereas HLA-II, CD326, CD19, CD20, CD25, ROR1, SSEA-4, HLA-I, and CD41b discriminated controls from patients with AMR. The diagnostic performance of each marker was reliable (AUROC: 0.727–0.939) [113]. Subsequently, the random forest (RF) classification model was used to establish two diagnostic models for 11 differentially expressed EVs markers. The first double-layer RF model discriminated between CAR and non-rejection with an accuracy of 100%, and then distinguished the two types of rejection, which also provided a high performance with an accuracy of 95%. The second combined model classified patients in a single step (non-rejection vs. ACR vs. AMR), and all patients were correctly identified. It is reasonable to conclude that machine learning models serve as a diagnostic reference for clinicians in collaboration with histological pathologists, reducing the number of biopsies and enabling the selection of patients at a high risk of rejection for closer follow-up.

In conjunction with machine learning, omics also provides novel insights into PGD risk stratification, improving donor-recipient matching, organ acquisition and preservation strategies, and pre-transplantation management at the algorithm level. In a large retrospective study of 219 HTx recipients, proteomics Olink panels were utilized to analyze the serum collected before transplantation [112]. Four Olink panels (Cardiovascular II, Cardiovascular III, Inflammation, Immune Activation) quantified 354 distinct protein biomarkers. Univariate and multivariate logistic regression models were used to identify 6 proteins in the derivation set that were significantly associated with PGD. Of these, only CLEC4C remained associated with PGD in the validation set after Bonferroni correction (OR: 3.04, 95% CI 1.74–5.82, p = 2.8 × 10^–4^). Compared with RADIAL score (AUROC: 0.55), CLEC4C (AUROC: 0.66, p = 0.048) or combined with clinical covariates (AUROC: 0.69, p = 0.018) considerably enhanced PGD recognition ability [112, 116].

Another multi-center study using TMTs LC–MS demonstrated that plasma KLKB1 level was the most crucial predictor of PGD (AUROC: 0.6444, OR: 0.1959). Following PRDX2, TPM4 and MPO (AUROC: > 0.6), the increased expression of each marker markedly predict PGD [111]. In contrast to biomarkers alone, the classifier performance of the multivariate model with KLKB1 integrated with the patients’ clinical features significantly improved (p < 0.0001). This classifier for predicting moderate-to-severe PGD in a prospective cohort was verified; AUROC was 0.71, the sensitivity and NPV were excellent, and the specificity and PPV were low. However, whether these KLKB1 results represent all HTx patients remains to be determined and needs to be further verified in a larger cohort. Consequently, researchers have adopted pre-transplant clinical and protein markers to predict survival after transplantation, and multivariate logistic regression models identified 11 protein markers that productively predicted graft survival [110]. SERPINF2, coagulation factor IX, and low molecular weights kininogen enabled the independent prognostication of PGD (AUROC: > 0.6), and maintained their predictive ability for 1-year survival.

Urine proteomics combined with machine learning may further improve the ability of urine biomarkers to predict HTx prognosis. In a single-center retrospective study, capillary electrophoresis coupled with mass spectrometry (CE-MS) was leveraged to detect the urine proteome of HTx patients, and an extreme gradient boosting (XGBoost) algorithm based on a decision tree was applied to construct a differential CAV proteomics model [104]. In the derivation cohort and validation cohort, the confirmed 27 protein peptides were able to distinguish CAV, AUROC for identifying CAV was 0.83 (95% CI 0.75–0.91, p < 0.001) and 0.71 (95% CI 0.60–0.8, p = 0.001), respectively. The logistic model with multiple clinical risk factors, integrated discrimination improvement was 9.1% (95% CI 2.5–15.3, p = 0.005), and net reclassification was increased by 83.3% (95% CI 46.7–119.5, p < 0.001). Additionally, among the 27 peptides, type I collagen accounted for the largest proportion, which may facilitate the migration and proliferation of smooth muscle cells (SMCs) through integrin mediation. This indicates that SMCs proliferation exists in the CAV and is related to the main fibrosis component, the collagen I urinary peptide of the extracellular matrix (ECM). These results provide insights into the pathological processes of CAV and personalized therapeutic targets. Recently, a study employed urinary PROteomics in Predicting HEart Transplantation outcomes study (uPROPHET) database and CE-MS to identify urinary proteins and evaluate osteoporosis post-HTx. Combined with support vector modeling (SVM) with 94 support vectors and the radial basis kernel function, the obtained OSTEO18 contains 18 urinary peptides. In clinical cohort, it usefully identified osteoporosis (AUROC: 0.83, 95% CI 0.76–0.90) [109].

## Conclusion

**Fig. 1:**
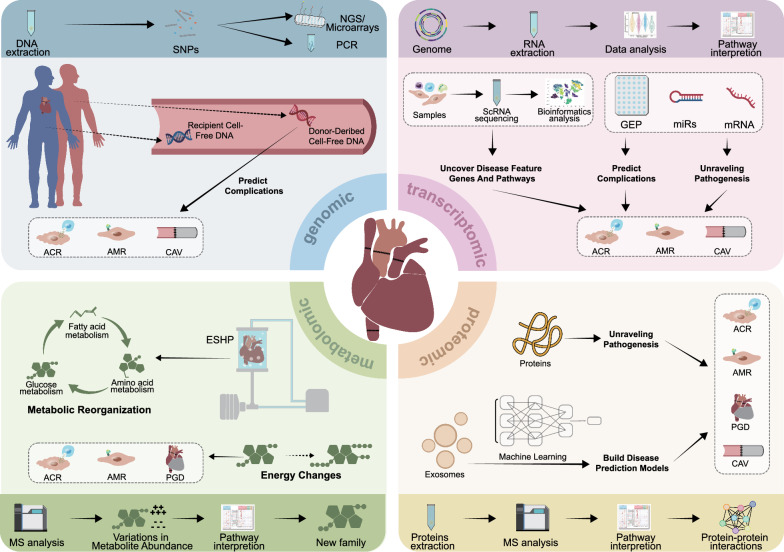
Leveraging multi-omics approaches in HTx. Multi-omics approaches including genomics, transcriptomics, proteomics, metabolomics, can be leveraged individually and via integration for biomarkers discovery to predict complications and unraveling pathogenesis, according to their specific analysis process as shown in the flow chart in the figure

This review systematically summarizes multi-omics associated investigations in HTx, including single-cell omics and machine learning integrated with omics (Fig. 1). We focused on novel non-invasive biomarkers discovered through various multi-omics techniques and their practical application capabilities in the clinical environment. On the other hand, we also concentrate on leveraging multi-omics to uncover the mechanisms underlying complications occurrence and progression, aiming to provide insights for novel targets design.

The next frontier in HTx omics research will focus on the following aspects: (1) Further exploration of accurate blood-based diagnosis to distinguish between ACR and AMR without the need for EMBx. (2) Further standardization of the clinical application protocol for the non-invasive combined monitoring of dd-cfDNA and GEP. (3) Advances in omics and single-cell technologies have revealed the importance of IFNs and IFN response genes in transplant rejection and CAV. The effectiveness of targeting the IFN signaling pathway needs to be evaluated to improve graft survival rates. (4) Based on the innovative biomarkers identified using omics, machine learning should be combined to construct risk stratification post-transplant to achieve personalized therapies. (5) Paying attention to the energy and metabolic changes in early or long-term graft dysfunction in HTx, and new pathophysiological mechanisms should be proposed. (6) It is necessary to conduct spatial transcriptomics and radiomics to evaluate the prognosis of HTx and assess biomarkers from multiple perspectives.

Consequently, multi-omics profiling using genomics, transcriptomics, proteomics, and metabolomics is increasingly used to monitor prognosis after HTx. While achieving accurate monitoring, the mechanisms of transplantation complications are analyzed, which effectively promote the personalized heart transplant care.

## Data Availability

Not applicable.

## Abbreviations

HTx: Heart transplantation
CAR: Cardiac allograft rejection
CAV: CAV
PGD: PGD
EMBx: Endomyocardial biopsies
ICA: Invasive coronary angiography
IVUS: Intravascular ultrasound
dd-cfDNA: Donor-derived cell-free DNA
ACR: Acute cellular rejection
AMR: Antibody-mediated rejection
ISHLT: International society for heart and lung transplantation
AST: American society of transplantation
GRAfT: Genomic research alliance for transplantation
SNPs: Single nucleotide polymorphisms
AUROC: Area under the receiver operating characteristic
NPV: Negative predictive value
PCR: Polymerase chain reaction
NGS: Next-generation sequencing
D‐OAR: Donor‐Derived cell‐free DNA-outcome alloMap registry
ddPCR: Digital droplet PCR
CMV: Cytomegalovirus
PPV: Positive predictive value
LC–MS: Liquid chromatography-mass spectrometry
GC/TOFMS: Gas chromatography/time-of-flight mass spectrometry
OR: Odds ratio
ESHP: Ex situ heart perfusion
TnI: Troponin I
TCHP: Temperature-controlled hypothermic preservation
SCS: Standard cold storage
NMR: Nuclear magnetic resonance
β-HB: β-Hydroxybutyrate
95% CI: 95% Confidence interval
GEP: Gene expression profiling
CARGO: Cardiac allograft rejection gene expression observational
PBMCs: Peripheral blood mononuclear cells
qRTPCR: Quantitative Real-Time PCR
IMAGE: Invasive monitoring attenuation through gene expression
HR: Hazard ratio
miRs: MicroRNAs
DSA: Donor-specific antibodies
NK: Natural killer
FFPE: Formalin-fixed paraffin-embedded
HLA: Human leukocyte antigen
IgG: Immunoglobulin G
UTR: Untranslated region
IL: Interleukin
TNF: Tumor necrosis factor
IFN: Interferon
LC–MS/MS: Liquid chromatography-tandem mass spectrometry
MMDx: Molecular microscope diagnostic
SOMAscan: Slow off-rate modified aptamer
mTOR: Mammalian target of rapamycin
DSP: Digital spatial profiling
TGF: Transforming growth factor
CHIP: Clonal genesis of indeterminate potential
DTRT: DNA-based transplant rejection test
EMIAGE: Extended molecular immunology for allograft gene expression
scRNA-seq: Single-cell RNA sequencing
TCR: T cell receptor
scTCR-seq: Single-Cell TCR sequencing
CFBs: Cardiac fibroblasts
CTLs: Cytotoxic T lymphocytes
MHC: Major histocompatibility complex
DAMPs: Damage associated molecular patterns
PRRs: Pattern recognition receptors
EVs: Extracellular vesicles
NTA: Nanoparticle tracking analysis
RF: Random forest
CE-MS: Capillary electrophoresis coupled with mass spectrometry
SMCs: Smooth muscle cells
ECM: Extracellular matrix
uPROPHET: Urinary PROteomics in predicting HEart transplantation outcomes study
SVM: Support vector modeling
